# Duodenal Perforation: Outcomes after Surgical Management at a Tertiary Care Centre—A Retrospective Cross-Sectional Study

**DOI:** 10.1155/2020/8392716

**Published:** 2020-10-28

**Authors:** Srinivas Bojanapu, Ronak Atulbhai Malani, Samrat Ray, Vivek Mangla, Naimish Mehta, Samiran Nundy

**Affiliations:** Department of Surgical Gastroenterology and Liver Transplantation Sir Ganga Ram Hospital, New Delhi 110060, India

## Abstract

**Introduction:**

Duodenal perforation is a common surgical emergency and carries mortality ranging from 4% to 30% reported in Western countries, but there is a paucity of reports from India. We aimed to determine the factors which influence the surgical outcomes in patients with duodenal perforation.

**Methods:**

We retrospectively analyzed prospectively collected data from January 2010 to December 2018.

**Results:**

A total of 55 patients were included in the study of which 69% (38) were males and 31% (17) were females (M : F = 4.5 : 2). The mean age was 52.3 years. The cause for duodenal perforation was duodenal ulcer (*n* = 25, 45.5%), followed by post-ERCP complications (*n* = 15, 27.3%), surgery (*n* = 11, 20%), and blunt trauma (*n* = 4, 7.2%) with perforations localized at D2 (*n* = 28, 51%) and at D1 (*n* = 27, 49%). Patients underwent primary repair with an additional diversion procedure (*n* = 28, 51%) and repair only in 18 (32.8%). There were 21 (38%) deaths. Patients with ERCP-associated duodenal perforation had longer hospital stay (*P* ≤ 0.001), ICU stay (*P*=0.049), duration of drainage (*P* ≤ 0.001), and higher leak rate (*P*=0.001) and re-exploration rate (*P*=0.037). A high mortality rate was seen in patients with preoperative organ failure (*n*  = 18, 78% versus 9.4%, *P*=0.001), postoperative leak (*n* = 7, 64% versus 32%, *P*=0.05), and longer duration from onset of symptoms to surgery (≥4 days) (*P*=0.045).

**Conclusion:**

Perforation of the duodenum is associated with high morbidity and mortality regardless of its cause and is higher in those who have a longer interval to surgery, preoperative organ failure, and a postoperative leak.

## 1. Introduction

Duodenal perforation is a common surgical emergency. It can be secondary to an ulcer, endoscopic procedure, trauma, or surgery for a non-gastroduodenal condition and carries a mortality rate ranging from 4% to 30% reported in Western countries. However, there are few reports comparing its outcome depending on the cause of the perforation.

It was previously a major complication of peptic ulcer (DU) disease; although it is now becoming progressively rarer with the increasing use of acid-lowering drugs, it still affects 2%–10% of such patients. Different authors have reported mortality rates in this condition ranging from 1.3% to 20% [1, 2]. It is also a feared complication of endoscopic retrograde cholangiopancreatography (ERCP), and in a review of 21 prospective studies, the incidence of post-ERCP duodenal perforation was 0.6%, and the perforation-related mortality was 9.9% [3]. Overall, 20% to 50% of these patients required surgery [4–6].

Trauma and abdominal surgery are other causes of duodenal perforation in 0.2%–3.7% of all trauma-related laparotomies, and the associated mortality of duodenal injuries was in the range of 11.2%–26% [7–9].

Advanced age, preoperative shock, coexisting medical illness, and delay in treatment are common risk factors associated with poor outcomes in patients with duodenal perforation [10]. We reviewed our experience of surgical management of the different causes of duodenal perforation to try and identify the predictors of outcome in such patients.

## 2. Patients and Methods

From a retrospective analysis of prospectively maintained data, we identified all patients who were operated for duodenal perforation admitted to our department at Sir Ganga Ram Hospital, New Delhi, between January 2010 and December 2018. Their demographic details, cause of perforation, preoperative variables such as organ failure, and the interval before surgery were collected. We used the Boey score to predict morbidity and mortality. For each patient, a score was assigned which was obtained by the total of three risk factors (medical illness, presence of shock defined by systolic blood pressure <90 mmHg or <60 mmHg of mean arterial pressure, and lag time defined as the duration of ulcer perforation to the presentation at the hospital). Intraoperative findings such as the site of the perforation and type of surgery were collected. Postoperative variables recorded included the total length of hospital stay, time in the intensive care unit (ICU), postoperative leak rate, need for re-exploration, postoperative complications (according to the Clavien–Dindo grades), duration of drainage, and in-hospital mortality.

For statistical analysis, we used the SPSS software (version 24) for Windows. The mean, median, and ranges were calculated for continuous variables. The chi-square test and Fisher's exact test were used to test the significance of the association between predictors of outcomes. Receiver-operating characteristic (ROC) curve analysis was used to estimate the predictive ability of the Boey score. The area under the ROC curve (AUC) indicates the probability of postoperative morbidity or mortality and actual postoperative condition and is considered perfect (1), good (>0.8), moderate (0.6–0.8), and poor (<0.6) for AUC. The level of significance was set as *P* < 0.05. The study has been reported in line with the STROCSS criteria [11].

## 3. Results

A total of 214 patients underwent surgery for duodenal diseases during this period, of which 55 patients underwent surgery for duodenal perforation.

### 3.1. Demographics

Of 55 patients with duodenal perforation, there were 38 males (69%) and 17 females (31%). Duodenal perforation was found to be significantly higher in males as compared to females (*P*=0.016). Their ages ranged from 16 to 81 (mean 52) years.

### 3.2. Aetiology

The most common cause for duodenal perforation was peptic ulcer (*n* = 25, 45.5%), followed by ERCP-associated perforations (*n* = 15, 27%), inadvertent injury during surgery for adjacent organs (*n* = 11, 20%), and trauma (*n* = 4, 7.3%). Of the 15 ERCP-associated perforations, two patients had come from elsewhere with perforation and two patients underwent delayed surgery due to failed conservative management. Of the eleven patients with duodenal perforations due to inadvertent duodenal injury, four (36%) were due to laparoscopic cholecystectomy, with missed intraoperative diagnosis and were referred from elsewhere to this institute. In the remaining six patients, duodenal perforation was only recognized at operation. Of the six patients, two (18%) perforations each occurred during open right hemicolectomy (for ileocaecal malignancy), right nephrectomy (for emphysematous pyelonephritis and xanthogranulomatous pyelonephritis), and pancreatic necrosectomy. One (9.1%) patient sustained duodenal perforation during the surgery for a jejunal enterocutaneous fistula. Blunt trauma was the cause of duodenal injury in four patients (Table 1).

### 3.3. Preoperative and Intraoperative Variables

23 patients (42%) had preoperative organ failure, thirteen had acute kidney injury (AKI) alone, six patients had AKI with respiratory insufficiency requiring respiratory support, and four patients had circulatory failure requiring inotropic support. However, none of the patients needed dialysis preoperatively. The mean duration between the onset of symptoms and surgery was 4.6 days (range 0–20), and the majority of perforations found were in the second part of the duodenum, i.e., D2 (*n* = 28, 51%) followed by D1 (*n* = 27, 49%). All patients had broad-spectrum antibiotics before surgical intervention, along with supportive measures. The most commonly performed procedure was a primary repair of the duodenal perforation with diversion, e.g., a gastrojejunostomy (*n* = 28, 51%), followed by primary repair of perforation only (*n* = 18, 33%), diversion only (*n* = 6, 11%), and a resectional procedure (*n* = 3, 5.4%). In all patients, wide-bore abdominal drains were placed. The decision to extubate or ventilate in the immediate postoperative period was taken in consultation with the critical care team (Table 1).

### 3.4. Outcome Variables

The mean length of hospital stay was 17 days (0–70), and the mean ICU stay was 5.9 days (0–28). Abdominal drains placed during the surgery drained for a mean duration of 12 days (0–70). Eleven patients (20%) developed a duodenal leak in the postoperative period, of which 6 (40%) were in the post-ERCP group, followed by 3 (27%) in the surgery group and one (8%) in the ulcer group. Of the 11 patients who developed a leak, five (45%) underwent re-exploration with lavage, drain placement, and a feeding jejunostomy, and we managed the remaining six patients conservatively. 22 (40%) patients had minor complications (Clavien grades I and II), and 12 (22%) had major complications (Clavien grades III and IV). Of the 55 patients, 21 patients (38%) died.

## 4. Association of Aetiological Variables with Outcome Variables

In patients who survived, the hospital stay was longer (>20 days) in those having duodenal perforation secondary to ERCP (n = 8, 53%) and with inadvertent duodenal perforation (n = 4, 36%) compared to patients with ulcer perforation. In this last group, there was no mortality (n = 0) (*P* < 0.001) (Table 2).

Post-ERCP perforation (*n* = 6, 40%) patients had a prolonged ICU stay (>10 days) as compared to those after ulcer (*n* = 1, 4%) and inadvertent injury group (*n* = 1, 9%) (*P*=0.049). Postoperative leaks were also significantly more common after ERCP (*n* = 6, 40%) as compared to ulcer perforations (*n* = 2, 8%) (*P*=0.001), and they also had a higher re-exploration rate (*n* = 4, 36%) compared to duodenal ulcer perforations (*n* = 0) (*P*=0.037). Post-ERCP perforation patients (*n* = 10, 66%) needed abdominal drains for longer periods (>10 days) compared to ulcer perforation (*n* = 3, 12%), postsurgery perforation (*n* = 2, 18%), and traumatic perforation (*n* = 1, 25%) (*P* < 0.001). However, no significant difference was observed among various aetiological variables for preoperative organ failure rate and mortality (Table 2).

### 4.1. Association of Mortality with Preoperative, Intraoperative, and Postoperative Variables

We also compared the association of mortality with various preoperative, intraoperative, and postoperative variables. Among all the factors studied, we found that age >50 years, duration to surgery (≥4 days), presence of preoperative organ failure, and postoperative leak to be significantly associated with mortality. Patients with age >50 years (*n* = 18, 62%) had higher mortality as compared to patients of ≤50 years of age (*n* = 3, 11.5%) (*P*=0.001). We found patients who underwent surgery ≥4 days (*n* = 16, 53%) had high mortality as compared to <4 days group (*n* = 5, 20%) (*P*=0.045). A higher mortality rate was observed in patients with preoperative organ failure (*n* = 18, 78%) compared to those without preoperative organ failure (*n* = 3, 9.4%) (*P* < 0.001). Similarly, mortality was higher in patients who had postoperative leaks (*n* = 7, 64%) as compared to those who did not have leaks (*n* = 14, 32%) (*P*=0.05). There was no significant association between mortality and gender, cause or location of the perforation, and type of surgery. On multivariate analysis, only preoperative organ failure positively correlated with mortality (*P*=0.002) (Table 3).

### 4.2. Boey Score

**Figure 1:**
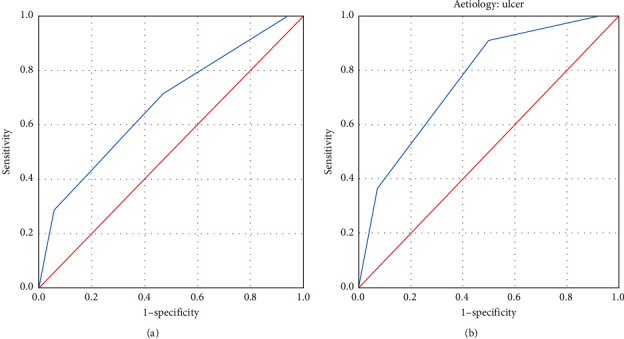
The area under the receiver-operating characteristic curve (AUC) for the (a) total group and (b) duodenal ulcer perforation group.

To predict morbidity and mortality, we used the Boey score. The majority of the patients had scores 1 (40%) and 2 (41%). Patients of peptic ulcer perforation had 50% (*n* = 6) and 80% (*n* = 4) mortality rates with scores of 2 and 3, respectively. However, in patients with ERCP-associated perforation, majority (46%, *n* = 7) had a score of 1, and mortality was at 40% (*n* = 2) in patients with a score of 2 (Table 4). ROC curve analysis demonstrated an AUC of 0.676 for the whole group and 0.766 for the duodenal ulcer perforation group (Table 5 and Figure 1).

## 5. Discussion

Duodenal perforation is commonly due to a peptic ulcer but is now changing with the progressive increase in the availability and usage of endoscopic, diagnostic, and interventional procedures. In addition, perforations are encountered due to inadvertent injury at an operation, for instance, adjacent or a retroperitoneal organ. Although there is a substantial literature on each of these causes, they are generally dealt with separately, and there are few reports to our knowledge comparing the outcomes of duodenal perforation from these different causes with each other mainly from the developing world.

We found that duodenal ulcer was found to be the most common aetiological factor leading to perforation and carried the best prognosis. Though the overall numbers of peptic ulcer perforations are not as expected in this part of the world, at the same time, we have a comparably higher number of ERCP and surgery-associated perforations that is due to the reason that most perforated peptic ulcer occurring in the low socioeconomic group generally seek treatment at public health care facility [12].

In this study, we observed that the mean age was 52.3 years, and the male : female ratio was 2.3 : 1.

However, few South Asian studies have reported a lower mean age of 40–43.4 years and a higher gender ratio of 10.5 : 1 [13, 14]. These studies were done exclusively on peptic ulcer perforations. The present study findings are comparable to that of Nuhu and Kassama in which they reported a mean age of 45.5 years, and the male: female ratio at 4.8 : 1 in the West African population [15]. The discrepancy could be due to the higher socioeconomic strata of patients seeking treatment at this institute and also an increase in the use of aspirin and anti-inflammatory medications in this age group.

Post-ERCP perforations were associated with a significantly prolonged hospital stay with a mean (±SD) of 26.9 (±21.4) days, ICU stay of 9.8 (±16.7) days, higher leak rate (*n* = 6, 40%), higher re-exploration rate, and prolonged requirement of keeping drain in situ as compared to other aetiological factors. It is explained with the fact that this group of patients required the addressal of the primary disease and additional procedures were done apart from addressing perforation. The findings are consistent with these studies [16–18]. However, we found no significant association in the mortality rate between the various aetiology groups.

In the inadvertent duodenal injury group, the majority (*n* = 4, 36%) occurred in the course of laparoscopic cholecystectomy, and none were recognized intraoperatively and were identified at a mean duration of 6.75 days (range 4–8) after the surgery. It is a well-recognized entity, and common duodenal perforation is secondary to a thermal injury with delayed manifestation. In a review of literature, the incidence of duodenal injury reported was 0.04% (range: 0.001%–4%) [19] and was generally identified during surgery or up to 5 days postoperatively in this study [20]. The associated mortality varied from 8.3 to 75%, as reported in a few studies [20], and in the present study, it is 18% (*n* = 2).

The Boey score is a well-validated scoring system for predicting morbidity and mortality and is used in patients with peptic ulcer perforation. In this study, patients with higher Boey score had higher mortality rate in all the groups of patients except the trauma group. Mortality occurred in 38% (*n* = 21) of the patients, but it climbed to 75% (*n* = 8) in patients with a Boey score of 3, and it was 39% (*n* = 23) in patients with a Boey score of 2. Although Boey et al. [21] developed this score for patients with peptic ulcer perforation, the score also associated well with mortality occurring in ERCP and surgery-related duodenal perforations. Though Boey et al. in 1987 reported 100% mortality in patients with a score of 3, the better outcomes in the present study can be attributed to the vast progress which has occurred in critical care management. The outcomes are similar to these published studies [22–25].

Boey et al. and Khan and Aziz, in their study, reported that delay in diagnosis and initiation of surgical treatment is associated with high morbidity and mortality after surgery [21, 26]. Although this was for duodenal ulcer perforation, in circumstances of ERCP-associated perforation, conservative management with diligent monitoring and subjecting them to surgery with failure of conservative therapy is also the standard of care. Conservative management is also equally efficacious in duodenal ulcer perforation [27]. However, it is vital to identify the duodenal perforation and stratify patients at the earliest to either conservative or surgical management.

Outcomes are generally poor when there is a delay in the diagnosis of perforation [28].

Delay in treatment, location of the perforation, advanced age, female gender, coexisting medical problems, failed primary surgery, and gastrectomy are the poor prognostic factors reported in the literature [29–32]. In the present study, gender, aetiology, location of the perforation, and type of surgery did not affect the mortality rate significantly.

Kim et al. found that age >60 years and female sex were associated with a high mortality rate [33], and in another study [25], though morbidity was higher in patients of >60 years , the mortality was higher in patients of <60 years. In our study age, > 50 yrs was associated with higher mortality, but the female sex was not associated with high mortality.

Studies have reported that delayed treatment, preoperative shock, and concurrent severe medical illness were the risk factors affecting prognosis [34, 35]. Early recognition and prompt intervention are paramount to reduce morbidity and mortality [21, 26]. Our study found a similar association of delayed surgical treatment and preoperative shock with higher mortality. In our study, age >50 years, the presence of preoperative organ failure, duration to surgery ≥four days, and postoperative leak were associated with a high mortality rate.

In the present study, mortality for duodenal perforation of all aetiologies was 38.2%. A significant proportion of patients presenting late (≥4 days) and with preoperative organ failure because of delayed referral could be the reason for such high mortality in our study.

Limitation: sample size.

## 6. Conclusion

Perforation of the duodenum is a common surgical emergency with a varied aetiology. We found that older age, the presence of preoperative organ failure, delayed presentation, and postoperative leak were factors associated with poor outcomes. Patients with higher Boey scores fare badly with high mortality rates across the aetiologies. Patients with ERCP-associated perforation, the second part of duodenum perforation, and those treated with repair and diversion, ICU stay, and longer duration of drains in situ had a significantly extended hospital stay but were not associated with higher mortality rates.

## Figures and Tables

**Table 1:** Demographic data, preoperative, intraoperative, and postoperative variables, and outcomes of duodenal perforation. Association of aetiological factors with outcome variables.

	*N*	Aetiology variables				
		Ulcer (%)	ERCP (%)	Postsurgery (%)	Trauma (%)	*P* value
*Total Patients*	55	25 (46 %)	15 (27%)	11 (20%)	4 (7.3%)	
*Age (years)*						
Mean	52.3	56.8	51	51.1	33	0.185
Range	16–81	25–75	29–81	23–69	16–48	
Male	38 (69.1%)	18 (72)	6 (40)	10 (90)	4 (100)	0.016
Female	17 (30.9%)	7 (28)	9 (60)	1 (10)	0	

*Indication for ERCP*	Choledocholithiasis	11 (73%)				
	Benign biliary stricture	3 (20%)				
	Malignancy	1 (7%)				

*Inadvertent injury during surgery*	Laparoscopic cholecystectomy	4 (36%)				
	Right hemicolectomy	2 (18%)				
	Pancreatic necrosectomy	2 (18%)				
	Right nephrectomy	2 (18%)				
	Enterocutaneous (jejunal) fistula	1 (9.1%)				

*Duration to surgery (days)*						
Mean	4.62	3.8	4.4	6.5	5.2	0.156
Range	0–20	1–12	0–20	0–20	1–13	

*Preoperative organ failure*						
Yes	23 (41.8)	11 (44)	7 (46)	2 (18)	2 (50)	0.320
No	32 (58.2)	14 (56)	8 (54)	9 (82)	2 (50)	

*BOEY score*						
	0 (3.6%)	1 (4%)	1 (6.6%)	0	0	
	1 (38%)	7 (28%)	7 (47%)	5 (46%)	2 (50%)	
	2 (42%)	12 (48%)	5 (33%)	4 (36%)	2 (50%)	
	3 (5.4%)	5 (20%)	2 (13.3%)	1 (9%)	0	

*Surgery*						
Repair	18 (33%)					
Repair + diversion	28 (51%)					
Diversion	6 (11%)					
Resection	3 (5.4%)					

*Location*						
D1	27 (49)	23 (92)	0	2 (18)	2 (50)	
D2	28 (51)	2 (8)	15 (100)	9 (82)	2 (50)	

*Hospital stay (days)*						
Mean	17					
Range	0–70					

*ICU stay (days)*		0.049				
Mean	5.9					
Range	0–28					

*Leak*		0.001				
Yes	11 (20%)					
No	44 (80%)					

*Re- exploration*		0.037				
Yes	5 (9.1%)					
No	50 (90%)					

*Drainage duration (days)*		0.001				
Mean	12.11					
Range	0–70					

*Complications*		0.8				
Clavien–Dindo III, IV	12 (22%)					
Clavien–Dindo I, II	22 (40%)					

*Mortality*		0.47				
Yes	21 (38%)					
No	34 (62%)					

**Table 2:** Association of aetiological factors with outcome variables.

Outcomes	Aetiology variables				*P* value
	Ulcer, *n* = 25 (%)	ERCP, *n* = 15 (%)	Surgery, *n* = 11 (%)	Trauma, *n* = 4 (%)	
*Hospital stay (days)*					

0–10	20 (80)	4 (27)	0 (0)	0 (0)	<0.001
11–20	5 (20)	3 (20)	7 (64)	3 (75)	
> 20	0 (0)	8 (53)	4 (36)	1 (25)	

*ICU stay (days)*					

0–5	19 (76)	6 (40)	8 (72)	1 (25)	0.049
6–10	5 (20)	3 (20)	2 (18)	2 (50)	
>10	1 (4)	6 (40)	1 (9.5)	1 (25)	

*Leak*					

Yes	2 (8)	6 (40)	3 (27)	0 (0)	0.001
No	23 (92)	9 (60)	8 (72)	4 (100)	

*Re-exploration*					

Yes	0 (0)	4 (36)	1 (9.1)	0 (0)	0.037
No	25 (100)	11 (63)	10 (90)	4 (100)	

*Drainage duration (days)*					

≤ 10	22 (88)	5 (33)	9 (81)	3 (75)	<0.001
>10	3 (12)	10 (66)	2 (18)	1 (25)	

*Preop organ failure*					

Yes	11 (44)	8 (53)	2 (18)	2 (50)	0.568
No	14 (56)	7 (47)	9 (82)	2 (50)	

*Mortality*					

Yes	11 (44)	6 (40)	2 (18)	2 (50)	0.479
No	14 (56)	9 (60)	9 (81)	2 (50)	

**Table 3:** Association of mortality with preoperative, intraoperative and postoperative variables, and multivariate analysis for factors affecting mortality.

	Mortality	
	Yes (*n* = 21, 38%)	No (*n* = 34, 62%)	*P* value
*Age*			
≤50 years (*n* = 26)	3 (11.5%)	23 (88%)	0.01
>50 years (*n* = 29)	18 (62%)	11 (38%)	

*Sex*			
Male (*n* = 38)	14 (37%)	24 (63%)	0.760
Female (*n* = 17)	7 (41%)	10 (59%)	

*Aetiology*			
Ulcer (*n* = 25)	11 (44%)	14 (56%)	0.479
Post-ERCP (*n* = 15)	6 (40%)	9 (60%)	
Postsurgery (*n* = 11)	2 (18%)	9 (82%)	
Trauma (*n* = 4)	2 (50%)	2 (50%)	

*Location*			
D1 (*n* = 25)	11 (44%)	14 (56%)	0.418
D2 (*n* = 30)	10 (33%)	20 (67%)	

*Duration to surgery*			
<4 days (*n* = 25)	5 (20%)	20 (80%)	0.045
≥4 days (*n* = 30)	16 (53%)	14 (47%)	

*Type of surgery*			
Repair only (*n* = 18)	8 (44%)	10 (56%)	0.927
Repair with diversion (*n* = 28)	10 (36%)	18 (64%)	
Diversion only (*n* = 6)	2 (33%)	4 (67%)	
Resection (*n* = 3)	1 (33%)	2 (67 %)	

*Preoperative organ failure*			
Yes (*n* = 23)	18 (78%)	5 (22%)	<0.001
No (*n* = 32)	3 (9.4%)	29 (90%)	

*Postoperative leak*			
Yes (*n* = 11)	7 (64%)	4 (37%)	0.05
No (*n* = 44)	14 (32%)	30 (69%)	

*Re-exploration*			
Yes (*n* = 5)	4 (80%)	1 (20%)	0.044
No (*n* = 50)	17 (34%)	33 (66%)	

*Multivariate analysis of factors affecting mortality*			
Age	0.071		
Organ failure	0.002		
Leak	0.412		

**Table 4:** Boey score with mortality in each aetiology group.

Boey score	Total, *n* = 55 (%)	Ulcer, *n* = 25	Death ulcer, *n* = 11 (%)	ERCP-associated, *n* = 15	Death ERCP-associated, *n* = 5 (%)	Surgery, *n* = 11	Death surgery, *n* = 2 (%)	Trauma, *n* = 4	Death trauma, *n* = 2 (%)
0	2 (3.6)	1	0	1	0	0	0	0	0
1	22 (40)	7	1 (14)	7	2 (28)	6	0	2	2 (100)
2	23 (41)	12	6 (50)	5	2 (40)	4	1 (25)	2	0
3	8 (15)	5	4 (80)	2	1 (50)	1	1 (100)	0	0

**Table 5:** The area under the receiver-operating characteristic curve (AUC) and 95% confidence interval of each aetiology of duodenal perforation.

Aetiology	Mortality (CI)
Whole group	0.67 (0.52–0.82)
Ulcer perforation	0.76 (0.57–0.95)
ERCP	0.56 (0.26–0.86)
Surgery	0.91 (0.71–1.0)
Trauma	0

## Data Availability

The data used to support the findings of this study are available from the corresponding author upon request.

## Additional Points

*New Knowledge Added by the Study*. (1) Comparison between outcomes of surgical intervention between different aetiologies. (2) The Boey score may be predictive of outcomes for ERCP-associated perforation. (3) Delayed surgery portends poor outcomes, with prolonged ICU and hospital stay. (4) Outcomes are not different from either first or second part of duodenal perforation. *Implications for Clinical Practice or Policy*. (1) The necessity to identify early and perform surgery prior to patient developing systemic decompensation. (2) Close monitoring in cases of ERCP-associated perforations to subject them for early surgery. (3) Helps in prognostication and information provided to the attendants.

## References

[1] Rajesh V., Chandra S. S., Smile S. R. (2003). Risk factors predicting operative mortality in perforated peptic ulcer disease. *Tropical Gastroenterology Official Journal of Digestive Diseases Found*.

[2] Hermans S., Christer S., Christer Staël M. (1999). Surgical approach and prognostic factors after peptic ulcer perforation. *The European Journal of Surgery*.

[3] Andriulli A., Loperfido S., Napolitano G. (2007). Incidence rates of post-ercp complications: a systematic survey of prospective studies. *The American Journal of Gastroenterology*.

[4] Polydorou A., Vezakis A., Fragulidis G., Katsarelias D., Vagianos C., Polymeneas G. (2011). A tailored approach to the management of perforations following endoscopic retrograde cholangiopancreatography and sphincterotomy. *Journal of Gastrointestinal Surgery*.

[5] Enns R., Eloubeidi M. A., Mergener K. (2002). ERCP-related perforations: risk factors and management. *Endoscopy*.

[6] Ercan M., Bostanci E. B., Dalgic T. (2012). Surgical outcome of patients with perforation after endoscopic retrograde cholangiopancreatography. *Journal of Laparoendoscopic & Advanced Surgical Techniques*.

[7] Allen G. S., Moore F. A., Cox C. S., Mehall J. R., Duke J. H. (1998). Delayed diagnosis of blunt duodenal injury: an avoidable complication. *Journal of the American College of Surgeons*.

[8] Bozkurt B., Özdemir B. A., Kocer B., Unal B., Dolapci M., Cengiz O. (2006). Operative approach in traumatic injuries of the duodenum. *Acta Chirurgica Belgica*.

[9] Testini M. (2003). Significant factors associated with fatal outcome in emergency open surgery for perforated peptic ulcer. *World Journal of Gastroenterology*.

[10] Dakubo J. C. B., Naaeder S. B., Clegg-Lamptey J. N. (2010). Gastro-duodenal peptic ulcer perforation. *East Afr Med J*.

[11] Agha R. A., Borrelli M. R., Vella-Baldacchino M. (2017). The STROCSS statement: strengthening the reporting of cohort studies in surgery. *International Journal of Surgery*.

[12] Rosenstock S. J., Andersen L. P., Rosenstock C. V., Bonnevie O., Jørgensen T. (1996). Socioeconomic factors in *Helicobacter pylori* infection among Danish adults. *American Journal of Public Health*.

[13] Arveen S., Jagdish S., Kadambari D. (2009). Perforated peptic ulcer in South India: an institutional perspective. *World Journal of Surgery*.

[14] Gupta S., Kaushik R., Sharma R. (2005). The management of large perforations of duodenal ulcers. *BMC Surgery*.

[15] Nuhu A., Kassama Y. (2008). Experience with acute perforated duodenal ulcer in a West African population. *Nigerian Journal of Medicine: Journal of the National Assocation Resident Dr Niger*.

[16] Langerth A., Isaksson B., Karlson B.-M., Urdzik J., Linder S. (2020). ERCP-related perforations: a population-based study of incidence, mortality, and risk factors. *Surgical Endoscopy*.

[17] Kim J. H., Yoo B. M., Kim J. H., Kim M. W., Kim W. H. (2009). Management of ERCP-related perforations: outcomes of single institution in Korea. *Journal of Gastrointestinal Surgery*.

[18] Alfieri S., Rosa F., Cina C. (2013). Management of duodeno-pancreato-biliary perforations after ERCP: outcomes from an Italian tertiary referral center. *Surgical Endoscopy*.

[19] Testini M., Piccinni G., Lissidini G. (2008). Management of descending duodenal injuries secondary to laparoscopic cholecystectomy. *Digestive Surgery*.

[20] Machado N. O. (2016). Duodenal injury post laparoscopic cholecystectomy: incidence, mechanism, management and outcome. *World Journal of Gastrointestinal Surgery*.

[21] Boey J., Choi S. K. Y., Alagaratnam T. T., Poon A. (1987). A prospective validation of predictive factors. *Annals of Surgery*.

[22] Lohsiriwat V., Prapasrivorakul S., Lohsiriwat D. (2009). Perforated peptic ulcer: clinical presentation, surgical outcomes, and the accuracy of the Boey scoring system in predicting postoperative morbidity and mortality. *World Journal of Surgery*.

[23] Menekse E., Kocer B., Topcu R. (2015). A practical scoring system to predict mortality in patients with perforated peptic ulcer. *World Journal of Emergency Surgery*.

[24] Score B. (2019). Predicting outcome in perforated peptic ulcer from tertiary referral center of Nepal. *ARC Journal of Surgery*.

[25] Agarwal A. (2015). Validation of Boey’s score in predicting morbidity and mortality in peptic perforation peritonitis in Northwestern India. *Tropical Gastroenterology*.

[26] Khan D. S. H., Aziz M. S. A. (2011). A review of 36 cases. *The Proffesional Medical Journal*.

[27] Larkin J. O., Bourke M. G., Muhammed A., Waldron R., Barry K., Eustace P. W. (2010). Mortality in perforated duodenal ulcer depends upon pre-operative risk: a retrospective 10-year study. *Irish Journal of Medical Science*.

[28] Wu H. M., Dixon E., May G. R., Sutherland F. R. (2006). Management of perforation after endoscopic retrograde cholangiopancreatography (ERCP): a population-based review. *HPB*.

[29] Bae S., Shim K.-N., Kim N. (2012). Incidence and short-term mortality from perforated peptic ulcer in Korea: a population-based study. *Journal of Epidemiology*.

[30] Chalya P. L., Mabula J. B., Koy M. (2011). Clinical profile and outcome of surgical treatment of perforated peptic ulcers in Northwestern Tanzania: a tertiary hospital experience. *World Journal of Emergency Surgery*.

[31] Rahman M. M., Islam M. S., Flora S., Akhter S. F., Hossain S., Karim F. (2007). Mortality in perforated peptic ulcer patients after selective management of stratified poor risk cases. *World Journal of Surgery*.

[32] Svanes C., Lie R. T., Svanes K., Lie S. A., Sørelde O. (1994). Adverse effects of delayed treatment for perforated peptic ulcer. *Annals of Surgery*.

[33] Kim J.-M., Jeong S.-H., Lee Y.-J. (2012). Analysis of risk factors for postoperative morbidity in perforated peptic ulcer. *Journal of Gastric Cancer*.

[34] Hill A. G. (2001). Management of perforated duodenal ulcer. Zuckschwerdt. https://www.ncbi.nlm.nih.gov/books/NBK6926/.

[35] Søreide K., Thorsen K., Harrison E. M. (2015). Perforated peptic ulcer. *The Lancet*.

